# Livestock Manure Type Affects Microbial Community Composition and Assembly During Composting

**DOI:** 10.3389/fmicb.2021.621126

**Published:** 2021-03-22

**Authors:** Jinxin Wan, Xiaofang Wang, Tianjie Yang, Zhong Wei, Samiran Banerjee, Ville-Petri Friman, Xinlan Mei, Yangchun Xu, Qirong Shen

**Affiliations:** ^1^Jiangsu Provincial Key Lab of Solid Organic Waste Utilization, Jiangsu Collaborative Innovation Center of Solid Organic Wastes, Educational Ministry Engineering Center of Resource-saving Fertilizers, Nanjing Agricultural University, Nanjing, China; ^2^Department of Microbiological Sciences, North Dakota State University, Fargo, ND, United States; ^3^Department of Biology, University of York, York, United Kingdom

**Keywords:** composting, physicochemical properties, microbial community, livestock manures, feeding habit

## Abstract

Composting is an environmentally friendly way to turn plant and animal wastes into organic fertilizers. However, it is unclear to what extent the source of animal waste products (such as manure) affects the physicochemical and microbiological properties of compost. Here, we experimentally tested how the type of livestock manure of herbivores (sheep and cattle) and omnivores (pig and chicken) influences the bacterial and fungal communities and physicochemical properties of compost. Higher pH, NO_3_-N, Total carbon (TC) content and C/N were found in sheep and cattle manure composts, while higher EC, NH_4_-N, Total nitrogen (TN) and total phosphorus (TP) content were measured in pig and chicken manure composts. Paired clustering between herbivore and omnivore manure compost metataxonomy composition was also observed at both initial and final phases of composting. Despite this clear clustering, all communities changed drastically during the composting leading to reduced bacterial and fungal diversity and large shifts in community composition and species dominance. While Proteobacteria and Chloroflexi were the major phyla in sheep and cattle manure composts, Firmicutes dominated in pig and chicken manure composts. Together, our results indicate that feeding habits of livestock can determine the biochemical and biological properties of manures, having predictable effects on microbial community composition and assembly during composting. Manure metataxonomy profiles could thus potentially be used to steer and manage composting processes.

## Introduction

Livestock manure has become one of the main causes of non-point source environmental pollution due to continuous expansion of livestock and poultry breeding industry (Sun et al., 2012). Correct treatment of livestock and poultry manures is thus required, which could be achieved through composting. Composting is an effective and widely used approach to transform animal manure and other agricultural waste products into high-quality agricultural organic fertilizers (Bernal et al., 2009). As a biological decomposition process, composting relies on the activity of a diverse set of microbes that are important drivers of the depolymerization of organic matter (Xi et al., 2015). Bacteria and fungi are the most abundant and active microbes involved in composting (Rastogi et al., 2020). While bacteria are responsible for a large proportion of decomposition and heat generation in compost (Lopez-Gonzalez et al., 2015), fungi are capable of decomposing complex polymers present in the compost (Yamamoto and Nakai, 2019). While the succession of microbial communities during different composting stages have been studied extensively (Nakasaki et al., 2005; Jiang et al., 2019), there is less information on how the initial manure metataxonomy composition affects composting. The initial compost metataxonomy composition is important because it affects the proliferation of mesophilic microbiota which are responsible for the rapid rise of composting temperature (De Gannes et al., 2013) and creation of a suitable environment for the secondary microorganisms during composting (Ryckeboer et al., 2003). Furthermore, initial composting microbiota could have long-lasting effects for later phases of composting, affecting the maturation and stabilization of composts (Villar et al., 2016). Therefore, information on the initial manure metataxonomy profiles could potentially be used to predict and manage the composting process.

While the activity, metabolism and abundances of microbes is heavily affected by the physicochemical properties of compost (Lopez-Gonzalez et al., 2015; Awasthi et al., 2017; Duan et al., 2019), also the used source materials play an important role important as most of the decomposing microorganisms originate from the raw materials. Of the physicochemical composting properties, temperature is an important indicator for the maturation of compost and reflects microbial activities during composting (Tang et al., 2007; Bernal et al., 2009). Temperature also affects microbial abundances and it has been found that fungi are likely more sensitive to fluctuating temperature and moisture contents than bacteria (Zhang et al., 2011). C/N ratio also reflects the degradation of organic matter by bacteria and fungi (Meng et al., 2019) and the initial C/N ratio can affect the microbial community structure (Lei and Vandergheynst, 2000; Zhang et al., 2011). It is, however, still unclear how different physicochemical compost properties shape the composition and assembly of bacterial and fungal communities, depending on the type of raw materials used for composting.

Livestock manures is one the common raw materials used in composting and microbial communities of livestock and poultry manures have been explored extensively during composting (Awasthi et al., 2019; Chen et al., 2019; Bello et al., 2020). It has been established that different composting systems using manure from different animals are associated with specific microbiota. For example, Firmicutes often dominate in pig manure compost (Wang et al., 2018b), while Proteobacteria are more abundant in cattle manure (Wang et al., 2018a). In case of fungi, Ascomycota and Basidiomycota have been found to be the core phyla in chicken and cow manures (Neher et al., 2013). While such variations could be driven by the composting process, it could also be due to initial differences in manure microbiota composition, determined by the diet of and gut microbiota composition of animals (Hussein et al., 2017; Chen et al., 2019). For example, previous studies have shown that the bovine feed contains more crude fiber, while chicken feed has higher contents of proteins and antibiotics (Sapkota et al., 2007; Chibisa et al., 2016). In general, carnivores consume food with significantly higher protein content than herbivores (Wilder et al., 2013). Such drastic diet differences will likely have consequences for the manure microbiota composition (Carmody et al., 2015), and gut microbiomes of herbivores have been shown to be more diverse compared to omnivores (Ley et al., 2008). While the animal feeding habits are likely to play an important role in determining the microbial composition of their manure, it is not clear if these differences are retained, or if they potentially have effects on the composting process.

Here, we studied this experimentally by comparing the bacterial and fungal communities of four different types of manures (sheep, cattle, pig, and chicken) at the initial and final stages of composting using a trough fermentation approach in the one factory. These four types of manures were selected because sheep and cattle are herbivores, and pig and chicken are omnivores, and hence have significant differences in their diets. Specifically, we investigated the link between physiochemical compost properties and bacterial and fungal communities using correlative analyses. We hypothesized that microbial communities should cluster according the feeding habits of animals, resulting pairing between the herbivore (sheep and cattle) and omnivore (pig and chicken) manure compost treatments. Moreover, we expected that such clustering would be clearer at the initial phase of composting, while community assembly could lead to either clearer divergence or convergence between manure compost treatments during composting.

## Materials and Methods

### Composting Process and Sampling

The composting experiments were carried out using a trough composting system at Senhe industry (120.3°E, 30.42°N), Hangzhou, Zhejiang, China, from May to July of 2018 with a period of 90 days. Four types of livestock manures (sheep, cattle, pig, and chicken) were collected from surrounding farms by Senhe industry and were used as main composting materials. All manures were amended with sawdust to adjust the moisture content to optimal at 65% (Richard et al., 2002). The properties of sawdust were following: Bulk density 0.29, Total porosity 77.5 g/cm^3^, Density 0.33 g/cm^3^, Gas-water ratio 0.79, pH 4.78, EC 0.73, and C/N 50.8. Composting was conducted in triplicate for each manure, and the dimension of each composting pile was 2.7 m × 4 m × 1 m (length × width × height). The compost piles were turned around periodically once a week. The temperature of each composting pile was recorded at the depth of 40 cm at 10:00 am and 4:00 pm every day. Samples were also collected at the depth of 40 cm from 10 separate places within each replicate composting pile and mixed evenly to obtain a representative composite sample. Samples were obtained for each type of manure compost at the beginning (days 0) and in the end (day 90) of composting. Samples were then divided into two aliquots, one preserved at −80°C for subsequent DNA extraction, and the other preserved at 4°C for analyzing the physicochemical compost properties.

### Analysis of Physicochemical Compost Properties

The pH and EC were measured using pH meter (E-201C, Shanghai, China) and conductivity electrode (DJS-1C, Shanghai, China) after shaking samples for 30 min (mixed with deionized water at1:10 wet weight of compost samples/water ratio). NH_4_-N and NO_3_-N contents were determined by extracting compost samples with 2 M KCl solution (1:10 ratio), and quantified using Nessler’s reagent colorimetry and spectrophotometry (Wang et al., 2015). TC and TN contents were measured with an Elementar Analysensysteme GmbH (Elementar Americas Inc., Hanau, Germany) using dry combustion, and the TC and TN contents were used to calculated C/N ratio (Cui et al., 2019). TP content was determined Agilent 710 ICP – OES following previously published standard methods (Zhang et al., 2012).

### DNA Extraction and High-Throughput Sequencing

Genomic DNA extraction was carried out by using the Power Soil DNA Isolation Kit (Mo Bio Laboratories, Solana Beach, CA, United States) according to the manufacturer’s instructions. DNA concentrations were determined with NanoDrop 1000 Spectrophotometer (Thermo Fisher Scientific, United States). Bacterial and fungal community compositions were characterized with multiplexed MiSeq sequencing at Shanghai Biozeron Biological Technology Co., Ltd (Shanghai, China). For bacteria, the primer pairs 563F and 802R that amplifies the V4 region of bacterial 16S rRNA gene was used (563F, 5′-AYTGGGYDTAAAGV G-3′ and 802R, 5′-TACNVGGGTATCTAATCC-3′) (Cardenas et al., 2010). For fungi, ITS1 and ITS4 primers were used to amplify the fungal ITS region (ITS1F, 5′-CTT GGTCATTTAGAGGAAGTAA-3′ and ITS2R, 5′- GCTGCGTTCTTCATCGATGC-3′) (White et al., 1990; Gardes and Bruns, 1993).

Paired-end reads from the original DNA fragments were merged using FLASH which has been designed to merge paired-end reads when at least some of the reads overlap the read generated from the opposite end of the same DNA fragment. Paired-end reads were assigned to each sample according to the unique barcodes. Raw FASTQ files were first demultiplexed according to the barcode sequences information using the following criteria: reads were discarded if (a) the 250 bp reads were truncated at any site receiving an average quality score <20 over a 10 bp sliding window, or if the truncated reads were shorter than 50 bp, if (b) two nucleotides mismatched in primer matching or if reads had ambiguous characters, or if (c) only sequences that overlap longer than 10 bp were assembled. Sequencing reads were processed with the UPARSE pipeline (Macdonald et al., 2011) and Ribosomal Database Project (RDP) database (Margesin et al., 2011). Sequences with ≥97% similarity were assigned to the same OTUs. Chimeric sequences that were identified using both *de novo* and reference-based chimera checking methods were removed from the data (Caporaso et al., 2010). The raw sequencing data used for this analysis are deposited into the NCBI Sequence Read Archive (SRA) database (PRJNA675153).

### Statistical Analysis

Principal component analysis (PCA) was applied to reduce the dimension of the original variables with “vegan” packages in R (Oksanen et al., 2013). The physicochemical properties were Z-score normalized by subtracting the mean value of each property, and dividing that result by the SD (Jain et al., 2005). After that cluster analysis of physicochemical properties was preceded by PCA. Shannon diversity index was calculated using the “diversity” function in the Vegan package in R. Microbial community structure was examined using principal coordinate analysis (PCoA) and groups compared using unweighted UniFrac distance matrix (Lozupone and Knight, 2005). The bacterial and fungal abundance data were min-max normalized before analysis. Data were analyzed with ANOVA (SPSS v16.0.) and linear mixed models (R) where dependent variables (e.g., Shannon diversity) were explained by composting phase and manure types. Differences between samples of bacterial and fungal compositions were characterized by hierarchical clustering according to Bray-Curtis dissimilarity and R Cluster package (version 3.5.0). Linear discriminant analysis effect size (LEfSe) was applied to search for statistically different biomarkers within different types of composts between initial and final phases (Segata et al., 2011). To explore how the physicochemical properties of composts drive the bacterial and fungal metataxonomy, Spearman’s rank correlation and Redundancy analysis (RDA) were conducted. One-way and two-way analysis of variance (Duncan’s multiple range test) were used to compare mean differences between the treatments using SPSS v16.0. In all analyses, *P* value < 0.05 were considered as be statistically significant differences.

## Results

### Physicochemical Properties of Composts

**FIGURE 1:**
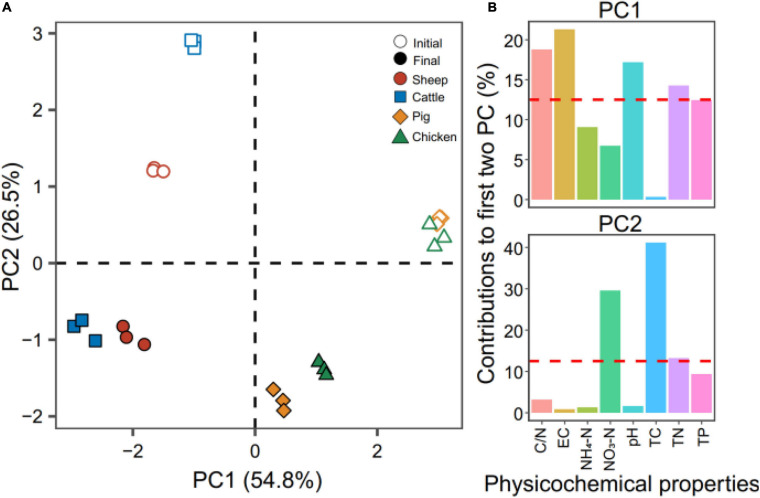
Principal component analysis of the physicochemical compost properties at the initial (open symbols) and final (filled symbols) phases of composting. In panel **(A)**, colors correspond to different types of manure used for composting. Panel **(B)** shows the contribution of each physiochemical property on the first two principal components in panel A that explained 81.3% of the total variation (54.8 and 26.5% for PC1 and PC2, respectively). Red dashed lines in panel **(B)** indicate the theoretical contribution of physicochemical properties. *N* = 3 for each type of manure compost.

The average differences in composting temperatures were compared by using the integral of the temperature time series “accumulated temperature.” The accumulated temperatures of pig and chicken manure composts were on average the highest, followed by sheep and cattle manure composts (*t*_5_ = −2.864, *p* = 0.035, Student’ s *t* test, Supplementary Figure 1). Principal component analysis revealed a strong effect of manure types on the physicochemical compost properties at the initial and final phases of composting (*R*^2^ = 0.99, *P* = 0.001, PERMANOVA test, Figure 1A). The first two principal components explained 54.8% (PC1) and 26.5% (PC2) of the total variation of the compost properties (Figure 1A). The PC was mainly explained by variation in EC (21.30%), C/N (18.75%), and pH (17.18%), while TC and NO_3_-N explained 41.16 and 29.55% of the variation on the second PC (Figure 1B). Higher pH, NO3-N, TC contents and C/N were found in sheep and cattle manure composts, while higher EC, NH_4_-N, TN, and TP contents were observed in pig and chicken manure composts both at the initial and final phase of composting (Supplementary Table 1). The significant decreases in TN and TC contents were consistently observed during composting regardless of the manure origin (Supplementary Table 1), and the variation in TN and TC contents also correlated with the changes in the C/N ratio. Together, these results suggest that the physicochemical compost properties varied across the four types of manure composts, but sheep and cattle composts, and pig and chicken composts were more similar to each other.

### Diversity and Structure of Microbial Communities

**FIGURE 2:**
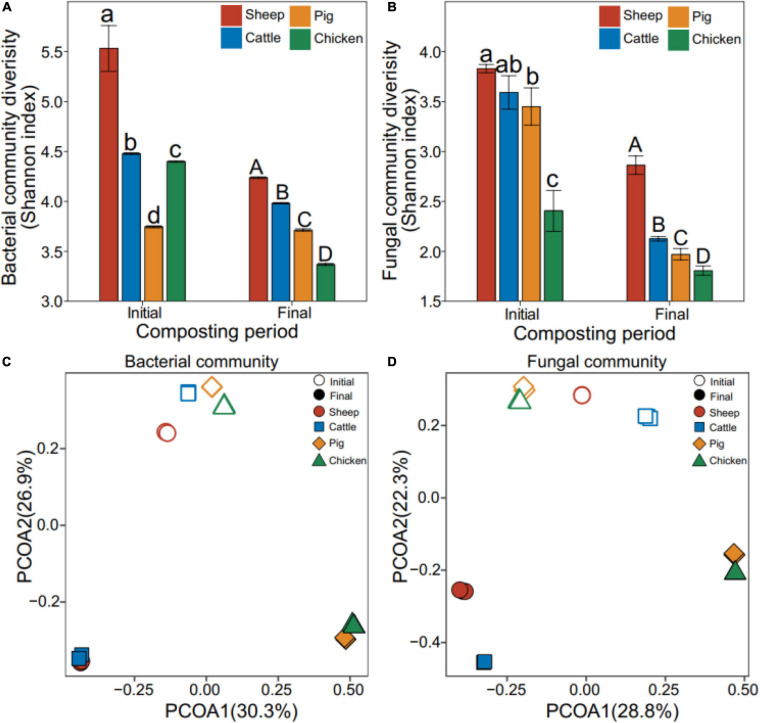
Effect of composting on manure compost metataxonomy diversity and composition. Shannon diversity of bacterial **(A)** and fungal **(B)** communities. Bacterial **(C)** and fungal **(D)** community composition based on the principal coordinate analysis of unweighted UniFrac distance matrix. Colors correspond to different types of manure composts, and open and closed symbols represented initial and final phases of composting, respectively. *N* = 3 for each type of manure composts.

The Shannon diversity of bacterial and fungal communities declined during the composting overall (bacteria: *F*_1,23_ = 10.7, *P* = 0.003; fungi: *F*_1,23_ = 29.1, *P* < 0.001, Figures 2A,B). While the Shannon diversity varied between manure compost types, the diversity of sheep and cattle manure composts were significantly higher compared to pig and chicken manure composts at both composting phases (bacteria: *F*_1,23_ = 12.5, *P* = 0.002; fungi: *F*_1,23_ = 6.07, *P* = 0.022, Figures 2A,B). In case of bacterial communities, community structures differed significantly between the initial and final composting phases (*R*^2^ = 0.99, *P* = 0.001, PERMANOVA, Figure 2C). Similar to the manure compost physicochemical properties, sheep and cattle composts, and pig and chicken composts had more similar bacterial communities both at the initial and final phases of composting, the similar pattern for pig and chicken manure composts (Supplementary Figure 2). Similar large shifts in fungal community composition were also observed during composting (*R*^2^ = 0.99, *P* = 0.001, PERMANOVA, Figure 2D). Moreover, the sheep and cattle manure composts were relatively more similar to each other, while pig and chicken manure composts grouped into one cluster (Supplementary Figure 2). Our results showed that composting drove bacterial and fungal community assembly to two different directions: while composting made sheep and cattle, and pig and chicken compost bacterial communities more similar to each other, fungal communities showed similar paired clustering both at the initial and final phases of composting.

### Composition of Microbial Communities

**FIGURE 3:**
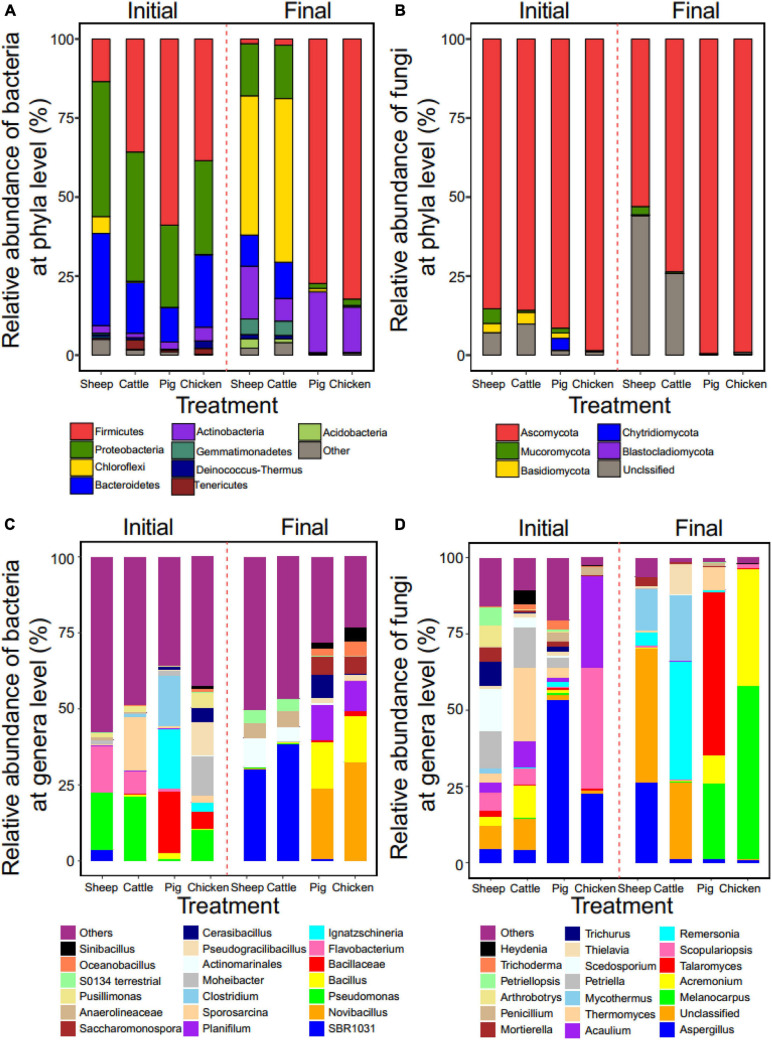
Effect of composting on manure compost metataxonomy at phyla and genera levels. The relative abundance of the dominant bacterial **(A)** and fungal **(B)** taxa at the phyla level in all manure compost treatments at the initial and final phases of the composting (based on OTUs of top 9 phyla). The relative abundance of the dominant bacterial **(C)** and fungal **(D)** taxa at the genera level (top 20 genera included). In all panels, colors correspond to relative abundance of different phyla or genera. *N* = 3 for each type of manure compost.

In case of bacterial communities, Firmicutes, Proteobacteria and Bacteroidetes were the predominant phyla. However, their abundances varied depending on the manure compost type at the initial phase of composting, where they clustered to sheep-cattle and pig-chicken pairs based on phyla abundances. The relative abundance of Proteobacteria in sheep (43%) and cattle (41%) manure composts, while the abundance of Firmicutes was higher in pig (59%) and chicken (38%) manure composts (Figure 3A). Similar patterns were also found at the end of composting but big changes in the dominant taxa was observed. Chloroflexi increased in sheep (44%) and cattle (51%) manure composts and became the new predominant phylum replacing Proteobacteria. In contrast, Firmicutes retained and increased in their relative abundance in pig (77%) and (82%) chicken manure composts. Actinobacteria increased their relative abundance during composting in all manure compost treatments (Figure 3A). In case of fungal communities, Ascomycota phyla dominated in all manure compost treatments both at the initial and final composting phases; this was especially clear in pig and chicken manure composts with over 99% relative Ascomycota abundances at the final phase of composting (Figure 3B).

The microbial community profiles were more complex at the genera level. We also found that the sheep manure compost had a similar profile to the cattle manure compost. While, *Pseudomonas* was the predominant genus initially in both communities (18 and 21% for sheep and cattle manure composts, respectively), it was replaced by *Anaerolineae SBR1031* during the composting (30 and 38% for sheep and cattle manure compost, respectively; Figure 3C). In contrast, bacterial genera profiles of pig and chicken manure composts differed initially, but showed a more similar pattern at the final phase. Specifically, *Novibacillus* (23%, 33%), *Bacillus* (15%, 15%), and *Planifilum* (11%, 10%) were the most common genera in these two types of manure composts (Figure 3C). The relative abundances of the fungal genera differed more between four manure compost treatment. While *Aspergillus* was present in all manure composts, its relative abundance in pig (53%) and chicken (27%) manure composts was significantly higher initially compared to two manure composts (Figure 3D). The relative abundance of *Mycothermus* was higher in sheep (14%) and cattle (21%) manure composts, while *Melanocarpus* dominated in pig (25%) and chicken (57%) manure composts at the final phase of composting (Figure 3D). To compared the indicator groups of bacteria and fungi in different manure composts, LEfSe analysis was conducted to identify the groups that displayed the significant differences among different composts (shown in cladogram in Supplementary Figures 3,4). It was found that, *Pseudonocardiaceae* of Actinobacteria was the main bacterial indicator in pig manure compost, while *Aspergillaceae* of Ascomycota fungi was notable fungal indicator in sheep manure compost at the end of composting. These results suggest that while clear metataxonomy clustering was observed at the phyla level, more variation between manure treatments were observed at lower taxonomic levels.

### Correlation Between Microbial Communities and Physiochemical Properties

**FIGURE 4:**
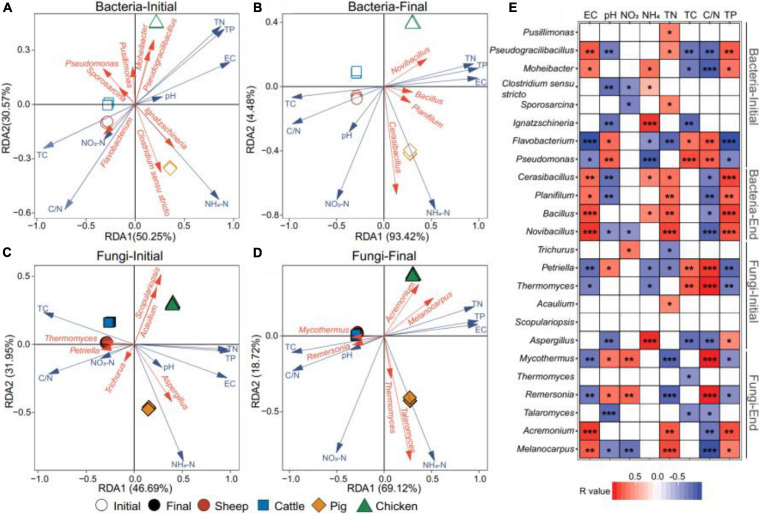
Correlations between physiochemical compost properties and microbial abundances based on redundancy analysis (RDA). Panels **(A,B)** show the relationship between bacterial and fungal genera with physicochemical composting properties (pH, EC, NH_4_-N, NO_3_-N, TC, TN, C/N, and TP) at the initial and final phases of composting. Panels **(C,D)** show the same relationship between fungal genera and physicochemical compost properties. In panels **(A–D)**, red vectors show the genera with >2% relative abundance, while blue vectors show compost physicochemical properties. Panel **(E)** shows Spearman correlation analysis between bacterial and fungal genera and compost physicochemical properties, where *r* > 0 denotes for positive correlations (red) and *r* < 0 for negative correlation s (blue); *, **, and *** denote for *P* values lower than 0.05, 0.01, and 0.001, respectively. *N* = 3 for each type of manure compost.

To investigate correlations between bacterial and fungal genera abundances with physicochemical properties of compost, a redundancy analysis (RDA) was conducted. We found that the first axes could explain 50.30 and 93.43% of the total variation of the bacterial genera abundances at the initial and final phase of composting (Figures 4A,B). In case of fungal communities, 46.71 and 69.14% of genera abundance variation was explained by the first two axes (Figures 4C,D). The Monte Carlo test was used to evaluate the impacts of physicochemical compost properties on microbial abundances. The EC, TN, and TP content had positive, and TC content and C/N ratio had negative effects on both bacteria and fungi at the initial and final phases of composting (Figures 4A,D and Supplementary Table 2). In line with previous analyses, physiochemical compost properties had similar effects on bacterial and fungal communities of sheep and cattle manure composts on both axes. However, pig and chicken manure composts responded similarly only along the first axis and more separation was observed along with the second axis. We also explored the relationships between the dominant bacterial and fungal genera with physicochemical properties of composts (Figure 4E). The abundance of *Pseudogracilibacillus* was positively correlated with the temperature, EC and TP content, while the abundance of *Flavobacterium* was negatively correlated with these parameters at the initial composting phase. There were positive relationships between the abundance of four genera (*Cerasibacillus*, *Planifilum*, *Bacillus*, and *Novibacillus*) and TN and TP contents at the final composting phase, which indicates that these bacteria caused or responded to changes in TN and TP contents. With fungal communities, the abundance of *Petriella* and *Thermomuces* were positively correlated with C/N ratio at the initial composting phase, while *Mycothermus* and *Remersonia* showed similar positive correlation at the final composting phase (Figure 4E). Together, these results suggest that the physiochemical manure compost properties strongly correlated with composition and assembly of bacterial and fungal communities during composting.

## Discussion

In this study, we investigated the link between the type of livestock manure, physiochemical compost properties and microbial community composition and assembly. We expected that microbial communities should cluster according the feeding habits of animals. In support for this, we found that both physical compost properties and microbial communities clustered closely depending if the manure originated from herbivores (sheep and cattle) or omnivores (pig and chicken). While clear reduction in bacterial and fungal diversity, and drastic changes in community composition, were observed during composting, microbial communities still retained clustering based on animal feeding habitats. Together, our results suggest that the type of manure used in composting raw materials is strongly linked with both physicochemical compost properties and microbial community composition and assembly during composting.

Based on our analysis, the physicochemical properties of different livestock manure composts clustered into two groups: herbivores (sheep and cattle) and omnivores (pig and chicken). As most nutrients ingested by animals are excreted into feces (Yano et al., 1999), the physicochemical properties of manure used in composting raw materials can reflect the feeding habits of livestock. As a result, observed differences were likely due to the animal dietary as herbivores consume more crude fiber in roughage-based diets, while omnivores have more crude proteins in their diets (Chen et al., 2019). The higher content of TN in pig and chicken manure composts was likely due to high protein contents in their feed (Chen et al., 2019), while higher TC contents and C/N ratios observed in sheep and cattle manure composts could be indicative of the presence of cellulose substances or unutilized complex nitrogen substrates in their feed (Pan et al., 2012). Relative higher TP content in pig and chicken manures was likely due to high phosphorus contents present in commercial feed to meet poultry’s phosphorus requirements (Yano et al., 1999; Sharpley and Moyer, 2000), while phosphorus contents were lower in herbivore manure composts. Animal feeding habits thus clearly affected the physicochemical properties of manure composts. Interestingly, while clear changes were observed during the composting, manure compost treatments still clustered according to herbivory and omnivory at the final phase of composting. This suggest that initial physicochemical differences between manure composts can persist throughout the composting.

Similar to physicochemical composting properties, bacterial and fungal metataxonomy clustered to two groups based on animal diets: herbivores (sheep and cattle) and omnivores (pig and chicken). Previous studies have showed that the differences in diets can affect the gut microbiome composition (Carmody et al., 2015; Hussein et al., 2017; Chen et al., 2019), possibly resulting in different composition of microbial communities in the manure of herbivores and omnivores. While microbial diversity decreased during composting, sheep and cattle manure compost retained significantly higher bacterial and fungal diversity compared to pig and chicken manure composts. These results are consistent with previous studies found that fecal microbiota of herbivores had a higher microbial diversity compared to omnivores and carnivores (Ley et al., 2008). Our analyses also revealed that the composition of both bacterial and fungal communities shifted considerably during composting in all manure compost treatments. However, paired clustering between herbivores (sheep and cattle) and omnivores (pig and chicken) was retained based on Bray-Curtis distances. Our data thus suggests that relative initial differences in manure compost community composition remained similar throughout the composting, and that selection by the composting conditions (Villar et al., 2016) was not strong enough to drive converging of different manure compost communities.

Sheep and cattle manure composts were initially dominated by Proteobacteria that encompass a huge morphological and physiological diversity, playing a critical role in the global cycles of carbon, nitrogen and sulfur (Spain et al., 2009). Interestingly, Chloroflexi became the most dominant phyla in these communities, which suggest that they had selective advantage over Proteobacteria during composting. In contrast, Firmicutes initially predominated in pig and chicken manure composts and increased in their relative abundance during composting. This could be explained by ability to form endospores that can help Firmicutes to survive high temperatures and harsh environment, which has also been shown in previous studies (Hartmann et al., 2014; Bello et al., 2020). At the genera level, bacterial community profiles in pig and chicken manure composts differed initially but converged toward the final phase of composting. This suggest that also the composting conditions were important in determining bacterial community assembly.

In case of fungi, Ascomycota dominated in all manure treatments during the whole composting process, which is in line with previous reports (Zhang et al., 2015; Akyol et al., 2019). One explanation for this is that Ascomycota can secrete a variety of cellulase and hemicellulase enzymes and hence can easily grow during composting by degrading organic materials available (Singh et al., 2003; Meng et al., 2019). The relative abundance of other two phyla, Mucoromycota and Basidiomycota, was initially higher in herbivore compared to omnivore manure composts and they both have been shown to play a vital role in decomposition of organic matter (Li et al., 2020) and straw residue (Ma et al., 2013). However, it is unclear why they were replaced by other species by the final phase of composting. At the genera level, *Aspergillus* dominated in pig and chicken manure composts initially. This could have been due to their innate ability to resist to bactericidal antibiotics, which are typically observed at high concentrations in pig and chicken manure (Liu et al., 2016). At the final composting phase, the relative abundance of *Mycothermus* was higher in sheep and cattle manure composts, while *Melanocarpus* dominated in pig and chicken manure composts. *Mycothermus*, a thermophilic genus, was reported can produce amylases, cellulases, lipases and xylanases (Aquino et al., 2003; Basotra et al., 2016), while *Melanocarpus* is able to produce alkaline active thermostable xylanases (Saraswat and Bisaria, 1997) that are important for degrading cellulose and hemicelluloses. Their ability to drive composting process thus likely explain increase in their relative abundances. Moreover, the four types of fermented manure composts which were dominated by different microbiota can be used as microbial inoculant to regulate the soil microbial community that promoting crop growth or stress resistance.

Previous research has reported that the bacterial and fungal communities are influenced by various physiochemical properties of composts (Duan et al., 2019). In our study, RDA analysis revealed that both the compositions of bacterial and fungal communities were significantly affected by NH_4_-N and TP contents, suggesting that compost metataxonomy were involved in the nitrogen and phosphorus metabolism. Pearson correlation showed that the abundance of *Ignatzschineria* and *Aspergillus* were positively correlated with NH_4_-N content, while *Cerasibacillus*, *Bacillus*, and *Novibacillus* positively correlated with TP content. *Ignatzschineria*, a Gram-negative genus, has been found in aerobic composts (Wang et al., 2017), but its function in the microbial community remains unclear. The success of *Aspergillus* could be due to its ability to utilize a huge range of nitrogen sources (Krappmann and Braus, 2005), while *Cerasibacillus*, *Bacillus*, and *Novibacillus* were possibly involved in the biodegradation of organic matters during composting (Wang et al., 2020) and potential P solubilization and mobilization via release of carboxylates (Arif et al., 2017; Saeid et al., 2018). These results indicated that both bacteria and fungi were sensitive to the changes of physiochemical properties of composts.

## Conclusion

The diversity and composition of bacterial and fungal communities before and after composting were distinct and clustered into two groups based on animal feeding habits: herbivores (sheep and cattle) and omnivores (pig and chicken) manure composts. Despite identical composting conditions, relative differences in community composition did not change much between different manure treatments. Furthermore, the shifts in both bacterial and fungal communities were closely related to physicochemical changes in compost properties, which could be mainly attributed to TN and TC contents of manure composts. Together, our results suggest that the initial physicochemical and biological properties of manure can predict the compost metataxonomy composition and assembly, potentially affecting the composting process efficacy and outcome. This study offers a new insight on how livestock manures with different physiochemical and microbiological properties could potentially be used steer and manage composting process and outcome on an industrial scale.

## Data Availability Statement

The original contributions presented in the study are publicly available. This data can be found here: NCBI Sequence Read Archive (SRA) database and accession number is PRJNA675153.

## Author Contributions

YX and ZW designed the experiments. JW conducted the experiments. XW and TY conducted the data analysis and wrote the manuscript with ZW, SB, and V-PF. All authors read and approved the final manuscript.

## Conflict of Interest

The authors declare that the research was conducted in the absence of any commercial or financial relationships that could be construed as a potential conflict of interest.
