# Supplementary material for: Livestock Manure Type Affects Microbial Community Composition and Assembly During Composting
Source: Front Microbiol. 2021 Mar 22;12:621126. doi: 10.3389/fmicb.2021.621126 (PMC8019744; doi:10.3389/fmicb.2021.621126)
Supplement: Supplementary file 1 [file Data_Sheet_1.docx]

Supplementary Material

**Livestock manure type affects microbial community composition and assembly during composting**

Jinxin Wan^1,^†, Xiaofang Wang^1,^†, Tianjie Yang^1^, Zhong Wei^1^, Samiran Banerjee^2^, Ville-Petri Friman^1,3^, Xinlan Mei^1^, Yangchun Xu^1,^*, Qirong Shen^1^

^1^ Jiangsu Provincial Key Lab of Solid Organic Waste Utilization, Jiangsu Collaborative Innovation Center of Solid Organic Wastes, Educational Ministry Engineering Center of Resource-saving fertilizers, Nanjing Agricultural University, Nanjing 210095, Jiangsu, Peoples R China

^2^ Department of Microbiological Sciences, North Dakota State University, Fargo, North Dakota, USA

^3^ University of York, Department of Biology, Wentworth Way, York, YO10 5DD, UK

† These authors have contributed equally to this work and share first authorship.

**^*^** Corresponding author: Prof. Yangchun Xu**;** Email: ycxu@njau.edu.cn

Tel: +86-025-84396824


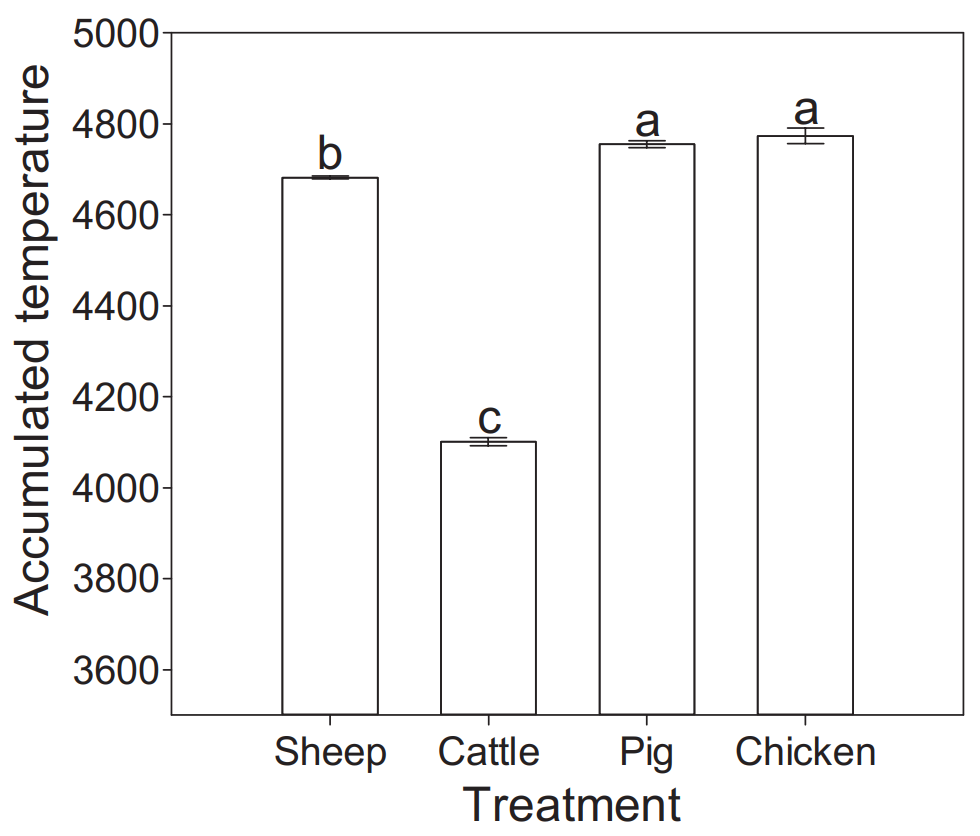


**Supplementary Figure 1|** The average temperature during 90 days of composting in different livestock manure compost treatments, which was defined as the integral area under the temperature curve as ‘accumulated temperature’. N=3 for each type of manure composts.


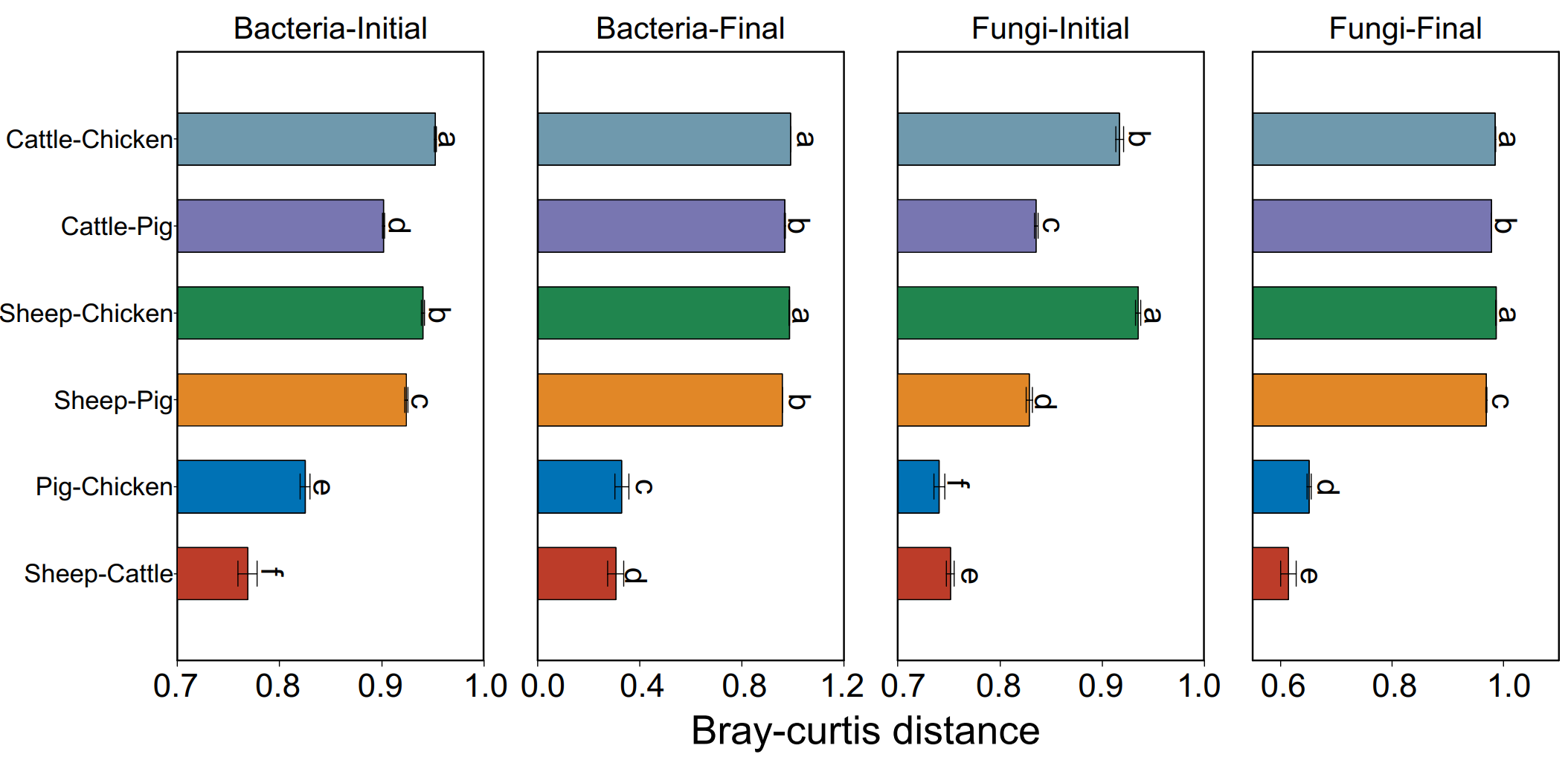


**Supplementary Figure 2|** The Bray-Curtis distances for bacterial and fungal communities between different types of composts at the initial and final phases of composting, respectively. Lowercase letters in all panels denote significant differences between treatments (Duncan’s multiple range test, *P* < 0.05). Error bars indicate the SD of the mean (N=9 for each pair).


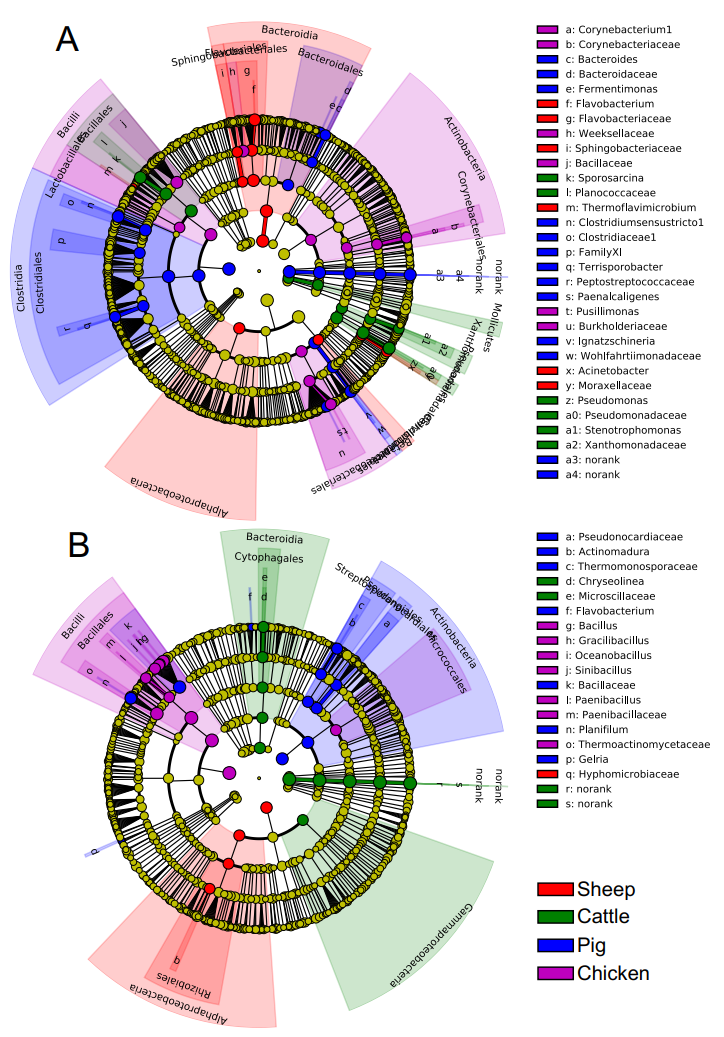


**Supplementary Figure 3|** The differential bacterial and fungal phylogenetic distribution for four manure composts treatment at the initial and final phases of composting. Panels show bacterial communities at the initial (A) and final (B) phases of composting. The linear discriminant analysis scores of ≥4 were used. Circles indicate phylogenetic levels from phyla to genera and each circle diameter is proportional with the taxon’s abundance. The nodes with different colors represent microbes that played an important role at different types of livestock manure composts, while yellow color represent non-significant taxa.


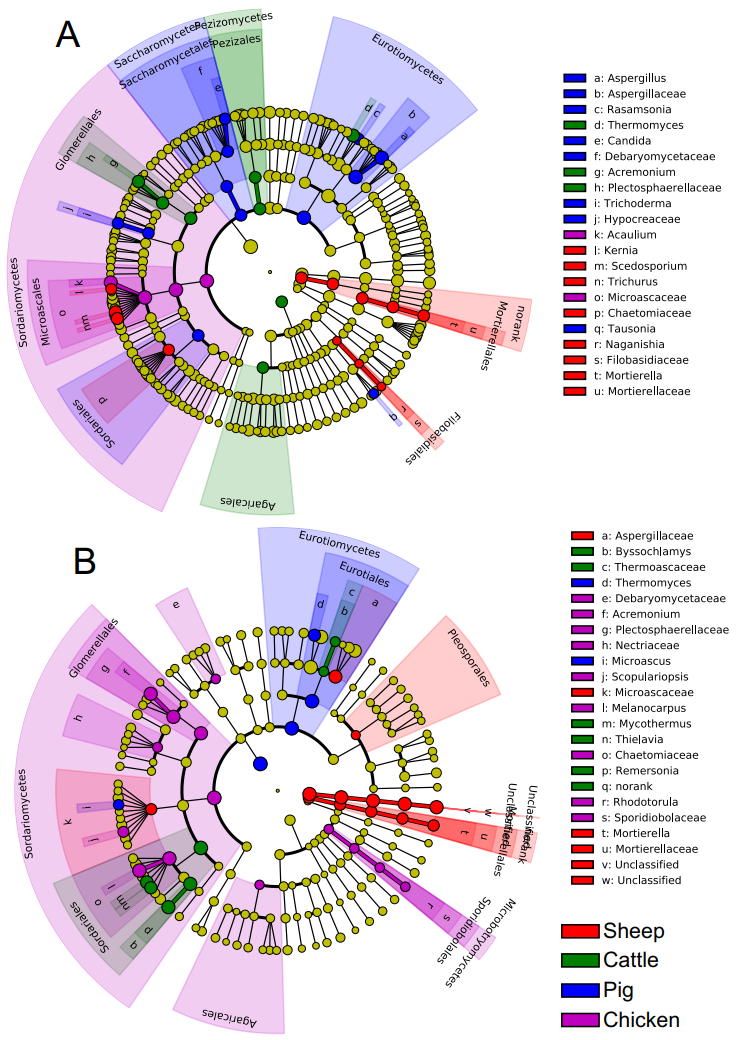


**Supplementary Figure 4|** The differential fungal phylogenetic distribution for four manure compost treatments at the initial and final phases of composting. Panels show fungal communities at the initial (A) and final (B) phases of composting. The linear discriminant analysis scores of ≥4 were used. Circles indicate phylogenetic levels from phyla to genera and each circle diameter is proportional with the taxon’s abundance. The nodes with different colors represent microbes that played an important role at different types of livestock manure composts, while yellow color represent non-significant taxa.

**Supplementary Table 1|** Physicochemical properties of four types of manure composts

| **Parameters** | **Initial phase (day 0)** | | | |  | **Final phase (day 90)** | | | |
| --- | --- | --- | --- | --- | --- | --- | --- | --- | --- |
|  | Sheep | Cattle | Pig | Chicken |  | Sheep | Cattle | Pig | Chicken |
| pH | 8.32±0.23a | 7.50±0.53b | 6.32±0.11c | 7.35±0.09b |  | 7.73±0.27B | 8.01±0.40A | 6.98±0.35D | 7.38±0.17C |
| EC (s/m) | 2.14±0.24c | 2.21±0.13c | 7.16±0.48a | 5.73±0.41b |  | 2.16±0.07C | 2.62±0.13B | 6.88±0.34A | 7.13±0.36A |
| NH_4_-N (g/Kg) | 0.62±0.01c | 0.52±0.01d | 8.06±0.07a | 1.30±0.06b |  | 0.13±0.01C | 0.31±0.02B | 1.01±0.10A | 0.98±0.07A |
| NO_3_-N (g/Kg) | 0.17±0.01a | 0.17±0.01a | 0.14±0.01b | 0.11±0.01c |  | 1.01±0.03B | 1.47±0.16A | 0.32±0.02C | 0.38±0.02C |
| TN (g/Kg) | 29.67±0.76c | 38.77±0.70b | 38.36±0.46b | 48.29±0.0.47a |  | 17.84±0.78C | 18.16±1.25C | 26.08±0.22B | 32.94±0.34A |
| TC (g/Kg) | 508.80±4.26b | 785.50±5.68a | 440.52±7.39c | 444.98±20.41c |  | 321.55±7.45C | 415.58±9.21A | 330.94±2.60C | 355.16±7.68B |
| C/N | 17.15±0.43b | 20.26±0.27a | 11.48±0.16c | 9.21±0.36d |  | 18.03±0.43B | 22.93±1.14A | 12.69±0.42C | 10.78±0.32D |
| TP (g/Kg) | 3.82±0.20d | 5.88±0.07c | 10.03±0.15b | 14.53±0.31a |  | 4.24±0.05D | 5.97±0.15C | 13.59±0.16B | 17.57±0.13A |

Notes: lowercase letters indicate significant differences between four composting treatment (manure type) at initial phase and capital letters indicate significant differences at the final phase (*p* < 0.05).

**Supplementary Table 2|** Monte Carlo permutation test for RDA of physicochemical properties on microbial communities.

|  | **RDA1** | **RDA2** | **R^2^** | ***P*** |  | **RDA1** | **RDA2** | **R^2^** | ***P*** |
| --- | --- | --- | --- | --- | --- | --- | --- | --- | --- |
|  | **Bacteria-Initial** | | | |  | **Bacteria-Final** | | | |
| EC | 0.972 | 0.236 | 0.981 | **0.004** |  | 0.964 | 0.266 | 0.963 | **0.001** |
| pH | 0.988 | 0.152 | 0.077 | 0.692 |  | -0.770 | -0.638 | 0.213 | 0.349 |
| NO_3_-N | -0.852 | -0.523 | 0.139 | 0.474 |  | -0.366 | -0.931 | 0.760 | **0.009** |
| NH_4_-N | **0.851** | **-0.525** | **0.999** | **0.001** |  | 0.729 | -0.684 | 0.925 | **0.012** |
| TN | 0.895 | 0.446 | 0.943 | **0.001** |  | 0.930 | 0.369 | 0.891 | **0.001** |
| TC | -0.968 | -0.252 | 0.922 | **0.005** |  | -0.958 | -0.288 | 0.925 | **0.001** |
| C/N | -0.776 | -0.630 | 0.843 | **0.001** |  | -0.903 | -0.429 | 0.977 | **0.001** |
| TP | 0.908 | 0.418 | 0.993 | **0.001** |  | **0.951** | **0.311** | **0.999** | **0.001** |
|  | **Fungi-Initial** | | | |  | **Fungi-Final** | | | |
| EC | 1.000 | 0.029 | 0.991 | **0.002** |  | 0.975 | -0.220 | 0.977 | **0.001** |
| pH | 0.972 | -0.236 | 0.110 | 0.604 |  | -0.932 | -0.362 | 0.212 | 0.358 |
| NO_3_-N | -0.848 | -0.529 | 0.130 | 0.527 |  | -0.777 | -0.629 | 0.741 | **0.016** |
| NH_4_-N | **0.729** | **-0.684** | **0.997** | **0.001** |  | 0.332 | -0.943 | 0.875 | **0.012** |
| TN | 0.971 | 0.239 | 0.955 | **0.002** |  | 0.992 | -0.127 | 0.912 | **0.001** |
| TC | -0.999 | -0.034 | 0.944 | **0.002** |  | -0.979 | 0.206 | 0.945 | **0.001** |
| C/N | -0.870 | -0.493 | 0.835 | **0.001** |  | -0.999 | 0.040 | 0.982 | **0.001** |
| TP | 0.975 | 0.223 | 0.995 | **0.002** |  | **0.987** | **-0.162** | **0.997** | **0.001** |
